# Comparative gene expression analysis of *Fas* and related genes in preeclamptic and healthy women: A cross-sectional study

**DOI:** 10.18502/ijrm.v13i4.6886

**Published:** 2020-04-30

**Authors:** Zaima Ali, Saba Khaliq, Saima Zaki, Hafiz Usman Ahmad, Khalid Pervaiz Lone

**Affiliations:** ^1^Department of Physiology and Cell Biology, University of Health Sciences Lahore, Lahore, Pakistan.; ^2^Department of Physiology, Lahore Medical and Dental College Lahore, Lahore, Pakistan.; ^3^Department of Obstetrics and Gynecology, Jinnah Hospital Lahore, Lahore, Pakistan.

**Keywords:** *Preeclampsia, TNF-α*, Fas, * Apoptosis.*

## Abstract

**Background:**

Preeclampsia is a hypertensive disorder of pregnancy affecting about 2-10% pregnancies worldwide. mRNA expression of tumor necrosis factor alpha *(TNF-α), Fas,* and *FasL* have been reported to be altered in placental bed in preeclamptic pregnancies. We hypothesized that the expression of these genes is also altered in peripheral blood mononuclear cells (PBMCs) in preeclampsia.

**Objective:**

To compare the expression of Fas receptor and related genes in PBMCs of preeclamptic and normotensive pregnant women.

**Materials and Methods:**

A cross-sectional comparative study comprising of 18 cases and 18 controls was designed. 5 ml of venous blood was drawn and collected considering aseptic measures. Buffy coat was separated by centrifugation and stored at -20°C. Favor Prep total RNA Isolation Kit (Favorgen, Taiwan) was used for RNA extraction. The mRNA expression of *TNF-α, Fas,* and *FasL* was measured by real-time polymerase chain reaction in PBMCs in preeclamptic and normal pregnancies.

**Results:**

A significant increase in mRNA expression of *TNF-α, Fas,* and *FasL* (p ≤ 0.001) was observed in PBMCs of preeclamptic pregnancies compared to the control group (p ≤ 0.001). Moreover, a significant positive correlation was found between the *TNF-α* mRNA expression and *Fas* and *FasL* (p ≤ 0.001).

**Conclusion:**

The results lead to the conclusion that mRNA expression of *TNF-α, Fas,* and *FasL* in the maternal PBMCs is altered in preeclamptic pregnancies and might contribute to the pathogenesis of the disease.

## 1. Introduction

The outcome of a successful pregnancy is mainly dependent on placental sufficiency but any disorder resulting in poor placentation during pregnancy leads to preeclampsia (PE), restricted growth of the fetus and placental abruption (1, 2). PE is a gestational disorder, characterized by a rise in blood pressure and proteinuria commencing at more than 20wk of gestational age. It contributes a large share to the burden of maternal morbidity and mortality with an overall incidence of 2-10% worldwide (3). Normal placentation revolves around balanced angiogenesis and apoptosis. A number of studies have addressed disturbances in apoptosis in the placental bed in PE (4-6). *Tumor necrosis factor alpha (TNF-α)* secreted by macrophages and monocytes can induce apoptosis in vascular smooth muscle cells surrounding the spiral arteries as well as human trophoblast in the placental bed (7). Trophoblast cells secret TNF-α and ischemia of the placenta results in increased release of this pro-inflammatory cytokine (8, 9). In normal pregnancy, there is a shift of maternal immune response from T helper 1 towards T helper 2 response (10). This shift does not occurs in pregnancies complicated by PE with a resultant increase in the production of type 1 cytokines, e.g. TNF-α, IL-2, and Interferon gamma. All of these induce inflammation (11, 12).

Fas and FasL, members of TNF-α superfamily, are representative of the intrinsic pathway of apoptosis and are highly expressed on invasive extra-villous trophoblast cells as well as maternal lymphocytes. Abundant FasL expression on trophoblast was proclaimed to induce apoptosis of harmful activated maternal lymphocytes while the enhanced Fas expression on invasive trophoblast results in apoptotic death of these cells in the developing placenta (13). Many studies have reported the disturbance of the intrinsic pathway of apoptosis with differential expression of *TNF-α, Fas,* and *FasL* in placental bed in pregnancies complicated with PE and intrauterine growth retardation (IUGR) (5, 14, 15). The emerging concept that release of placental remains into maternal blood modulates the gene expression of circulating blood cells has persuaded the researchers to study gene expression in circulating blood cells (16). Moreover, cells outside the placental bed like macrophages and monocytes have been reported to produce TNF-α (7). We hypothesized that peripheral blood mononuclear cells (PBMCs) might contribute as an extra placental source of mRNA expression of *TNF-α, Fas,* and *FasL*. Only preliminary data is available regarding this extra placental source (PBMCs) of these inflammatory and apoptotic mediators. The mRNA expression of these genes in PBMCs in PE has not been studied by real-time polymerase chain reaction (PCR) except for *TNF-α* (17). The selection of PBMCs as the experimental tissue using real-time PCR as the study method is novel regarding these three genes expression analysis in PE. To elucidate the role of mRNA expression of these genes, a cross-sectional comparative study was designed.

The aim of this study was to evaluate and compare the mRNA expression of *TNF-α, Fas,* and *FasL* in maternal blood PBMCs in PE and healthy pregnancies in Pakistani women.

## 2. Materials and Methods

### Sample collection

The participants for this cross-sectional comparative study were collected from tertiary care hospital of Lahore from October 2016 to March 2017. Cases comprised of 18 pregnant women (between the ages of 18 and 40 yr) in the third trimester (28-40 wk), diagnosed as preeclamptic and 18 age-matched normal pregnant women at the same gestational age, considered as controls. The criteria for PE were: new onset of systolic blood pressure >140 mmHg or diastolic blood pressure ≥90 mmHg at >20 wk of gestation accompanied by 24 hr proteinuria ≥300 mg (≥1+ on dipstick), in at least two random urine samples collected 4-6 hr apart. Cases were further grouped into early-onset PE (EOP, 28-32 wk) and late-onset PE (LOP, 32.1-40 wk) with nine women in each group. All the participants were non-smoker. Women with a history of diabetes mellitus, renal disease, arthritis, inflammatory bowel disease, chronic hypertension, cardiovascular illness (e.g. ischemic heart disease), or other chronic inflammatory disease were excluded. Demographic data were recorded along with complete medical, obstetric, and family history.

5 ml of venous blood was drawn and collected considering aseptic measures in EDTA-coated vacutainer. Buffy coat was separated by centrifugation and stored at -20°C within an hour of sample collection. Favor Prep total RNA Isolation Kit (Favorgen, Taiwan) was used for RNA extraction following the manufacturer's instructions. The concentrations of extracted RNA were measured using Nano-drop and stored at -80°C in RNase/DNase free water.

### Quantitative real Time-PCR

**Figure 1:**
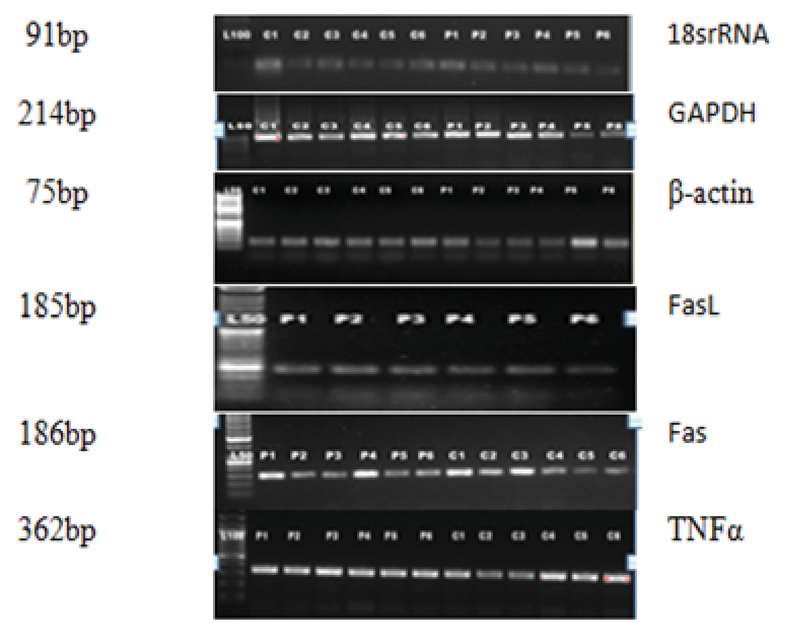
Confirmation of expected size products.

Two µg of total RNA of each sample was used to synthesize cDNA. RevertAid First-strand cDNA Synthesis kit (Thermo Scientific, USA) was used to synthesize cDNA following the manufacturer's instructions. Gene expression was quantified for 18 cases and 18 controls using synthesized cDNA and gene-specific-primers for Real-time PCR (Table I) on CFX 96 (Biorad, USA) using 2 X SYBR Green master mix (Fermentas, USA) according to the manufacturer's instructions. All reactions were carried in 20 µl of the reaction mixture with 1 µl of cDNA, 8 µl of 2 X SYBR Green Real-Time PCR Master mix, 0.5 µl of forward and reverse primers, and RNase-free water (Fermentas, USA). Real-time-PCR protocol was 94°C for 4 min, followed by 30 cycles of 94°C for 30 sec, annealing at 60°C for 30 sec, and extension at 70°C for 42 sec in a thermal cycle followed by melt curve analysis. Samples were assayed in duplicate and three housekeeping genes were used for data normalization. Relative gene expression analysis was performed using the 2-ΔΔ ct  method. Confirmation of the products of the expected size was done by electrophoresis on agarose gel (Figure 1).

**Table 1:** Sequence of primers used for quantitative real-time PCR


**Gene**	**Sequence**	**PCR product length (bp)**
*Fas-FP*	5'-CACCCCCAAACATGGAAATA-3'	
*Fas-RP*	5'-GGGTGGGGGAAAAATAAGAA-3'	186-bp
*FasL-FP*	5'-CTGGGGATGTTTCAGCTCTTC-3'		
*FasL-RP*	CTTCACTCCAGAAAGCAGGAC-3'	185-bp
*TNFα-FP*	5'-GGAGAAGGGTGACCGACTCA-3'		
*TNFα-RP*	5'-CTGCCCAGACTCGGCAA-3'	362-bp
*GAPDH-FP*	5'-ACG GAT TTG GTC GTA TTG GG-3'		
*GAPDH-RP*	5'-CGC TCC TGG AAG ATG GTG AT-3'	214-bp
*β-actin-FP*	5'-TCC ACC TTC CAG CAG ATG TG-3'		
*β-actin-RP*	5'-GCA TTT GCG GTG GAC GAT-3'	75-bp
*18s rRNA-FP*	5'-AGA AAC GGC TAC CAC ATC CAA-3'		
*18s rRNA-RP*	5'-CCT GTA TTG TTA TTT GTC ACT ACC T-3'	91-bp

### Ethical consideration

The study was performed in accordance with the ethical standards of the institutional ethical review (code: UHS/Education/126-16/2754) and with the Declaration of Helsinki 1964 and its later amendments. Informed written consent was obtained from all individual participants included in the study.

### Statistical analysis

Statistical analysis was performed using Statistical Package for the Social Sciences, Version 22.0, Armonk, New York, USA (SPSS). The clinical parameters were expressed as means ± SD (standard deviation). Expression of the studied genes was normalized against the mean of three housekeeping genes, that is, GAPDH, β-actin, and 18srRNA. Shapiro Wilk test was used to check the normality of data. The mRNA expression was compared using fold change values between two groups by students *t* test and One-way ANOVA with post-hoc Tukey's test for comparison between the multiple groups (EOP, LOP, and normotensive group). Differences in gene expressions were reported as fold change with SD. Pearson's correlation was used to find the relationship between the expressions of different genes. P ≤ 0.05 was considered statistically significant.

## 3. Results

### Clinical characteristics

The study population consisted of 18 women with PE and 18 healthy normotensive controls. Maternal ages of the participants were 28.2 ± 5 and 25.4 ± 3 for the case and control groups, respectively (p = 0.06). Similarly, the difference between the gestational age at the time of sampling in the case group (32.3 ± 3) was not significant from the controls (32.2 ± 3) (p = 0.91).

### Gene expression analysis

**Figure 2:**
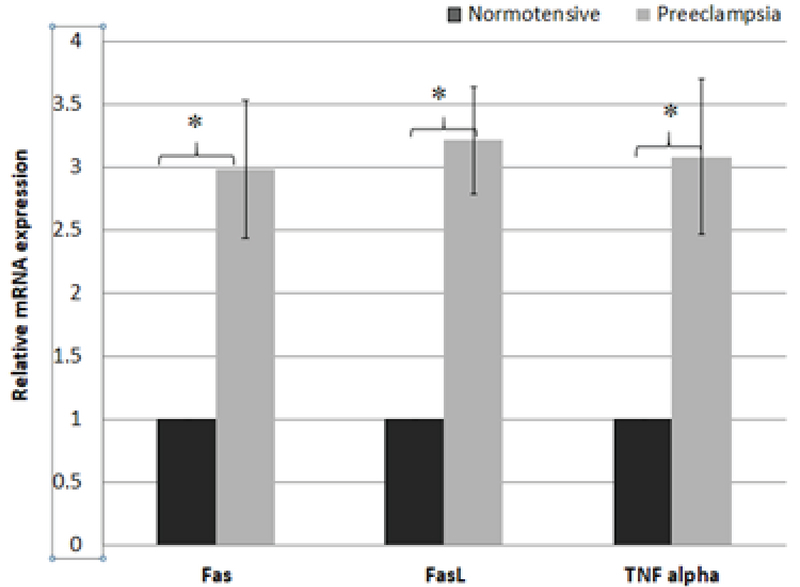
Comparison of PMBCs mRNA expression (fold change) between normotensive group and preeclampsia. Mean ± SD; *p < 0.001 analyzed by students' *t* test

**Figure 3:**
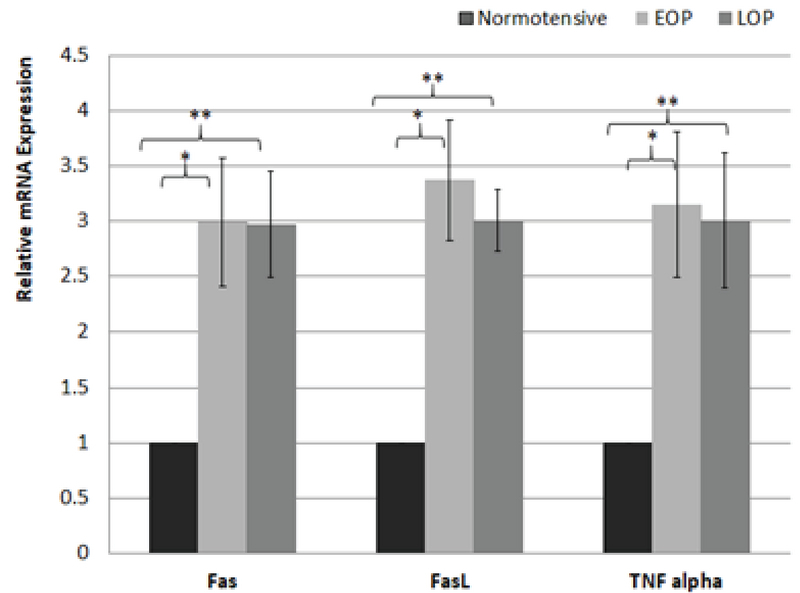
Comparison of PMBCs mRNA expression (fold change) between normotensive, early onset preeclampsia (EOP) and late onset preeclampsia (LOP). Mean ± SD; *p < 0.001 EOP vs normotensive; **p < 0.001 LOP vs normotensive, analyzed by ANOVA with post-hoc Tukey's test

Gene expression between different groups was reported as fold change (Figures 2, 3). The PBMCs mRNA expressions of *Fas, FasL* and *TNF-α* were significantly different between the case and control groups with a marked increase in the case group (p ≤ 0.001 for all). FasL showed a marked increase of 3.21-fold while the results for *TNF-α* and *Fas* were 3.08 and 2.98-fold, respectively (Table II).

For a detailed analysis of gene expression, the participants in the case group was divided into two subgroups, EOP and LOP, depending upon the time of onset of the disease. The difference in the mRNA expression of all the studied genes was not significant between the two subgroups (Table II).

### Correlations between *TNF-α, Fas,* and *FasL* mRNA expression

mRNA expression of *TNF-α* showed a positive correlation with Fas expression in the case group calculated by Pearson's correlation (Pearson's rho 0.95, p < 0.001). Similarly, FasL was found to be positively correlated with *TNF-α* (Pearson's rho 0.88, p < 0.001, Table III).

**Table 2:** Comparison of mRNA expression between different groups


**Gene**	**Normotensive (a)**	**Preeclampsia (b)**	**EOP (c)**	**LOP (d)**	**P-value**
*Fas*	1.00	2.98 ± 0.55	2.99 ± 0.58	2.97 ± 0.55	a & b: <0.001* c & d: 0.48** a & c: <0.001** a & d: <0.001**
*FasL*	1.00	3.21 ± 0.43	3.37 ± 0.48	3.01 ± 0.28	a & b: <0.001* c & d: 0.34** a & c: <0.001** a & d: <0.001**
*TNF-α*	1.00	3.08 ± 0.62	3.15 ± 0.66	3.00 ± 0.61	a & b: <0.001* c & d: 0.78** a & c: <0.001** a & d: <0.001**
Data are presented as Mean ± SD. EPO: early onset preeclampsia; LOP: late onset preeclampsia *P-value analyzed by students *t* test; **P-value analyzed by ANOVA with post-hoc Tukey's test					

**Table 3:** Correlation between *TNF-α* and *Fas, FasL* expression


**Gene**		**Pearson's rho (** ***r*** **)**	**P-value**
*TNF-α*	*Fas*	0.95	<0.001*
*TNF-α*	*FasL*	0.88	<0.001**
*P-value analyzed by Pearson's correlation test between TNF-α and Fas; **P-value analyzed by Pearson's correlation test between TNF-α and FasL			

## 4. Discussion

This study has reported elevated mRNA expression of *TNF-α* and its family members *Fas* and *FasL* in PBMCs in pregnancies complicated with PE as compared to normal pregnancies. The Fas-FasL, mediators of the intrinsic pathway of apoptosis, are deranged and show an incline in the expression in disorders characterized by disrupted immune tolerance or enhanced inﬂammation. Defective placentation with the disturbance in apoptosis in the placental bed has been well-studied. Researchers have reported differential Fas and Fas ligand expression in trophoblast in PE (5, 18). In 2011 Tomas and colleagues showed a disturbance of these with a decline in expression of FasL in the villous trophoblast in pregnancies complicated with PE (5). In contrast, Nishizawa and co-workers found an increase in FasL expression but no difference in Fas expression in placental tissue in PE and normal pregnancies (18).

In addition to trophoblast, Fas and FasL pathway is expressed and used by maternal lymphocytes. Altered expression of Fas has been reported in the lymphocytes in PE with a decrease in peripheral Gamma Delta T cells (γδ) and decidual Natural killer (NK) cells (19). This decrease in the expression of Fas suggests decreased susceptibility of these cells to apoptosis, increasing the possibility of their contribution to the pathogenesis of preeclampsia (19). Moreover, utero placental ischemia results in excessive shedding of syncytiotrophoblast micro particles into the maternal circulation in PE. These syncytiotrophoblast micro particles (STBM) accentuate the systemic inflammation by enhancing the expression of proinflammatory cytokines by PBMCs, enlightening their role in the widespread inflammation (12). We hypothesized that the Fas and FasL pathway is altered in the PBMCs in PE and reported an increase in mRNA expression of both *Fas* and *FasL*. In contrast, Kuntz and colleagues have reported a decrease in surface expression of these two genes on leukocytes and lymphocytes in PE (20). The variation in the results could be attributed to the difference in the time of sample collection. In our study sample collection was done in the third trimester while they took the samples after delivery. The levels of different inflammatory markers change from late pregnancy to the postpartum period (21).

Defective placentation in PE results in oxidative stress and increased production of proinflammatory TNF-α with resultant activation of leukocytes (22). In addition to increase in Fas and FasL in PE, we found a 3.08-fold increase in mRNA expression of *TNF-α* which is in accordance with the results reported by Chen and colleagues (17). Role of the excessive maternal systemic inflammatory response to pregnancy in the pathogenesis of PE is well established. There is a shift of cytokine profile in PE with an increase in proinflammatory cytokines as compared to the predominance of regulatory cytokine in normal pregnancy (23). A higher level of TNF-α, the proinflammatory cytokine, has been reported in the serum of pregnancies complicated with PE (24, 25). Extra placental sources like activated leukocytes or the endothelium might contribute to the increased levels of this inflammatory marker as there was no significant difference in the placental tissue (25). The higher mRNA expression of *TNF-α* in the PBMCs in PE in this study supports the involvement of tissues other than placenta in the pathogenesis of the disease. TNF-α enhances the production of eosinophil oxidants and toxicity toward human endothelial cells, activating the respiratory burst, and making the endothelium more vulnerable to oxidant damage (26). This study has also showed a significant positive correlation of TNF-α with Fas and FasL (p ≤ 0.001). Our results are supported by Fluhr and co-workers who reported that *TNF-α* up regulates the expression of *Fas* in human endometrial stromal cells, sensitizing them to apoptosis (27). Moreover, *TNF-α* is known to promote Fas expression in trophoblast cells as evident by an in vitro study by Aschkenazi and colleagues (28). They worked on trophoblast cell cultures and found increased sensitivity of these cells to Fas-mediated apoptosis after treatment with TNF-α. Serum from preeclamptic pregnancies decreased the viability of trophoblast cells and increased their sensitivity to Fas-mediated apoptosis in first-trimester trophoblast cell line (29). Our findings of increased expression of FasL in PBMCs, enlighten the probability that these cells might contribute to enhanced apoptosis of trophoblast expressing Fas. Excessive apoptosis of cytotrophoblast in placenta is reported by a number of researchers (30-32). Similarly, an increase in *Fas* expression by PBMCs in PE might result in apoptotic death of these cells by the *FasL*-expressing cytotrophoblast, affecting the survival of the fetal allograft escaping immune recognition.

## 5. Conclusion

The study concludes that mRNA expression of *TNF-α, Fas,* and *FasL* is altered in the maternal PBMCs in preeclamptic pregnancies and might contribute to the pathogenesis of the disease. Increased expression of *TNF-α, Fas, FasL* mRNA in PBMCs highlights the role of enhanced apoptosis and inflammation in PE. The regulation of *TNF-α* and *Fas, FasL* interactions in PBMCs need to be investigated in future approximations to better understand their role in the pathogenesis of PE. Longitudinal studies with multiple serial samples in different trimesters of pregnancy could enlighten the role of *Fas, FasL* pathway in the pathogenesis of PE. These processes can be potential targets for therapeutic agents to modify the course and improve the prognosis of the disease.

##  Conflict of Interest

The authors declare that there is no conflict of interest.
